# Association of dietary practice, rather than knowledge or attitude, with health-related quality of life in elderly NMIBC survivors: a cross-sectional study

**DOI:** 10.3389/fnut.2026.1918491

**Published:** 2026-08-27

**Authors:** Xiaodong Qing, Jiawen Huang, Wenbo Gao

**Affiliations:** 1 Department of Surgery, Ningbo Integrated Traditional Chinese and Western Medicine Hospital, Ningbo, Zhejiang, China; 2 Department of Urology, Ningbo Integrated Traditional Chinese and Western Medicine Hospital, Ningbo, Zhejiang, China

**Keywords:** bladder cancer, dietary knowledge-attitude-practice, health-related quality of life, intravesical chemotherapy, non-muscle-invasive bladder cancer

## Abstract

**Background:**

Health-related quality of life (HRQoL) is a critical concern in elderly non-muscle-invasive bladder cancer (NMIBC) survivors, but modifiable dietary factors remain underexplored. Using a cross sectional design, this study aimed to investigate the association between dietary knowledge-attitude-practice (KAP) scores and HRQoL in recurrence-free elderly NMIBC patients 12 months after TURBT, and to identify which KAP dimension shows the strongest independent association.

**Methods:**

We enrolled 106 patients (aged ≥60 years) recurrence-free at 12 months post-TURBT who completed intravesical therapy. Dietary KAP was assessed using a culturally adapted questionnaire, analyzed both continuously and dichotomized at the median (42) for descriptive purposes. HRQoL was measured with the FACT-Bl total and Trial Outcome Index (TOI). Multivariable linear regression adjusted for age, sex, education, smoking, tumor stage, and therapy type.

**Results:**

The High-KAP group (*n* = 53) had significantly higher FACT-Bl total (115.4 *vs.* 94.2) and TOI (84.1 *vs.* 68.5, both *p* < 0.001) as compared with Low-KAP group. In continuous analyses, each 1-point KAP increase was associated with a 0.58-point higher FACT-Bl (95% CI: 0.42–0.74) and 0.62-point higher TOI (0.48–0.76). Among subdomains, only practice remained significant (*β* = 1.24 and 1.31, both *p* < 0.001); while knowledge and attitude were not.

**Conclusion:**

Higher dietary KAP strongly is associated with better HRQoL, and this association appears primarily attributable to actual practice, not knowledge or attitude. These findings support behavior-focused, culturally tailored dietary support in survivorship care, although causality cannot be inferred due to the cross sectional design.

## 1 Introduction

Bladder cancer (BC) ranks as the ninth most prevalent malignant tumor globally, with approximately 75% of newly diagnosed cases classified as non-muscle-invasive bladder cancer (NMIBC) (1). Currently, transurethral resection of bladder tumor (TURBT) remains the standard primary treatment for NMIBC. However, these patients are challenged by a life-long risk of tumor recurrence and the burden of repeated cystoscopic examination and intravesical interventions, making the improvement of health-related quality of life (HRQoL) a critical clinical concern. Given the favorable long-term survival of most NMIBC patients, HRQoL has become an important indicator reflecting overall wellness rather than merely oncological outcomes (2). Nevertheless, disease-related symptoms and postoperative management could impose adverse impacts on patients’ daily functioning, and such unfavorable effects are more prominent among elderly survivors, who usually present with weaker physiological tolerance against treatment burdens (3). Thus, HRQoL is of particular concern in this aging population.

Elderly patients (aged ≥60 years) (4) are susceptible to deteriorated physical reserve and higher comorbidity risks, which could aggravate treatment-induced toxicities and weaken their psychological resilience toward recurrence monitoring. Meanwhile, advanced aging leads to unique dietary obstacles including polypharmacy-induced taste and appetite changes, chewing or swallowing dysfunction, as well as ingrained long-term eating habits. In this context, modifiable dietary factors measured by the knowledge-attitude-practice (KAP) framework have aroused growing research interest to recognize, understand, and facilitate improvement in their association with HRQoL, especially among aging BC survivors (5). The KAP framework was chosen over alternative models (e.g., Health Belief Model, Theory of Planned Behavior) because it explicitly separates knowledge, attitude, and practice as distinct measurable domains. This three-part structure allows us to identify which specific component rather than mere intention or perceived risk is most strongly associated with HRQoL. This question is particularly critical in older adults where habitual and functional barriers often prevent knowledge from translating into practice. However, most previous studies have focused on general dietary patterns or single nutrient, and few have simultaneously examined the three KAP dimensions in relation to disease specific HRQoL in older cancer survivors. Moreover, elderly individuals often face unique barriers such as polypharmacy, altered taste perception, chewing difficulties, and fixed eating habits that may weaken the translation of nutritional knowledge into actual behavioral change (6). Therefore, understanding which KAP component most closely correlates with HRQoL is essential for designing pragmatic, action-oriented interventions for this vulnerable population.

HRQoL comprehensively describes patients’ perceived life experience modified by disease impairment, functional status and social condition. For BC survivors, these factors are frequently compromised by tumor-related and treatment-related symptoms. Consequently, BC-specific complications frequent micturition, hematuria, urinary incontinence, dysuria, and persistent psychological anxiety over disease progression—can substantially disturb these domains (7). In clinical studies, multiple validated instruments are available for BC-related HRQoL assessment, covering generic instruments including the Medical Outcomes Study 36-Item Short Form (SF-36), and disease-specific questionnaires such as the European Organization for Research and Treatment of Cancer Quality of Life Questionnaire (EORTC QLQ-NMIBC24), Bladder Cancer Index (BCI) and Functional Assessment of Cancer Therapy Bladder Cancer (FACT-Bl) (8). As a mature self-reported dedicated tool for BC, FACT-Bl evaluates five core domains; and particularly, its composite Trial Outcome Index (TOI) precisely captures physical function and bladder-specific symptoms, rendering it sensitive to reflect HRQoL variations triggered by lifestyle adjustments like dietary modification (9).

Multiple domestic and international guidelines recommend intravesical Bacillus Calmette-Guérin (BCG) instillation for intermediate and high-risk NMIBC (10). However, intravesical chemotherapy with mitomycin C (MMC) or gemcitabine (Gem) dominates local clinical practice at our institution, due to several reasons, for example, regional BCG shortage, economic burden and patient preference. Under these real-world treatment regimens, impaired HRQoL continues to pose a persistent clinical challenge. Therefore, there is a clear need to explore additional modifiable lifestyle factors (11). Accumulating evidence indicates that dietary patterns exert independent effects on cancer prognosis and postoperative quality of life (12). Our prior case–control study additionally demonstrated that increased dietary KAP levels correlate with decreased long-term recurrence risk in patients with NMIBC (13). Nevertheless, relevant investigations focusing on the association between dietary KAP and HRQoL remain scarce, especially among elderly patients without recurrence 12 months after TURBT, a vital time node for routine postoperative follow-up. Accordingly, this real-world cross-sectional study aims to explore whether higher dietary KAP levels are associated with better HRQoL in elderly NMIBC survivors 12 months after TURBT.

## 2 Materials and methods

### 2.1 Study design and participants

This was a cross-sectional, single-center survey study conducted at Ningbo Integrated Traditional Chinese and Western Medicine Hospital (Zhejiang, China) between June 2025 and April 2026. Patients were recruited from the Department of Urology outpatient clinic during routine follow-up visits. We included patients who met all of the following criteria: (i) histologically confirmed NMIBC (including carcinoma *in situ* (CIS), Ta, or T1); (ii) at least 12 months after TURBT and confirmed recurrence free based on cystoscopy and imaging examinations according to standardized criteria; (iii) aged 60 years or older; and (iv) had completed a full course of intravesical instillation therapy as recommended by guidelines for their risk group (14). The 12-month period was chosen because it represents a critical time point for routine postoperative surveillance and completion of the initial induction/maintenance intravesical therapy, allowing assessment of mid-term survivorship outcomes. Patients were excluded if they had muscle invasive bladder cancer, upper tract urothelial carcinoma, other malignancy within the past 5 years, or cognitive impairment that would prevent valid questionnaire completion.

All participants provided written informed consent, and the study protocol was approved by the ethics committee of our hospital (approval No.: 2026–014). Out of 132 eligible patients recruited, 106 were eventually enrolled. The reasons for non-enrollment included: incomplete questionnaires (*n* = 11), refusal to participate (*n* = 9), and incomplete data with no imputation performed (listwise deletion, *n* = 6). Among the 106 enrolled patients, all had received intravesical therapy: 101 (95.3%) received intravesical chemotherapy (56 MMC, 45 Gem), and only 5 (4.7%) received BCG. No patient received both types of instillation.

Sample size consideration: Based on the rule of thumb for multiple linear regression (10–15 events per predictor variable), and given that our final model included 7 independent variables, a minimum of 70–105 participants was required. Our final sample of 106 patients therefore provides adequate statistical power to detect moderate-to-large effect sizes (anticipated *R*^2^ ≥ 0.30) with *α* = 0.05 and power = 0.80. No formal a-priori power calculation was performed due to the exploratory nature of the study.

### 2.2 Dietary KAP questionnaire

The dietary Knowledge Attitude Practice (KAP) questionnaire used in our study was adopted from our previously published case control study (13) which investigated the association between dietary patterns and late recurrence of NMIBC. For the practice domain, participants were asked to recall their usual dietary intake over the past 12 months (i.e., since their TURBT), consistent with the time-frame used in our previous validation study.

Briefly, the questionnaire comprises three dimensions: knowledge (11 items), attitude (8 items), and practice (8 food categories). The “knowledge” dimension uses 11 true/false/do not-know questions on dietary factors related to BC outcomes, such as the benefits of hydration and cruciferous vegetables, and the risks of preserved seafood, processed meats, and fried foods. Each correct answer scores 1 point, giving a knowledge score range of 0–11. The “attitude” dimension includes 8 Likert items (each score of 1–5) measuring perceived importance, self-efficacy, barriers, and willingness to modify diet, including local preserved seafood. The total attitude score ranges from 8 to 40. The “practice” dimension assesses actual intake of 8 core categories (e.g., water, cruciferous vegetables, preserved seafood, cooking methods for fresh fish, and tea) using a localized frequency format. Each item is scored on the basis of predefined favorable levels (e.g., water ≥1.5 L/day, cruciferous vegetables ≥2/week, preserved seafood <1/month), yielding a practice score of 0–16. Although the three domains have different numbers of items and score ranges, we calculated a total KAP score by summing the raw scores of each domain after confirming that each domain contributed roughly equally to the overall variance (Cronbach’s *α* = 0.91). To avoid over-interpretation of the total score, we also analyzed each subdomain separately in the main regression models. Thus, the overall KAP score ranges from 8 to 67.

The questionnaire was culturally tailored to the coastal Zhejiang population through several modifications. These included: (1) addition of regionally common preserved seafood items (salted yellow croaker, dried salted shrimp, fermented raw crab); (2) separate assessment of cooking methods for fresh fish (steaming/boiling versus deep frying/grilling); (3) replacement of generic vegetables with locally accessible cruciferous vegetables (bokchoy, gailan, bai cai); (4) use of local household measures (e.g., “one rice bowl,” “one small tea cup”) validated in a pilot survey of 30 local bladder cancer survivors; and (5) translation and back translation with input from five experts (two urologists, two clinical nutritionists and one nursing expert). The final questionnaire demonstrated good internal consistency (Cronbach’s *α* = 0.910) and face validity. Content validity was established through expert review. Construct validity was not formally tested via factor analysis due to the relatively small sample size, but the questionnaire’s items were derived from a literature-based framework and adapted to the local population through the pilot study (13).

For descriptive comparisons only, based on the median total KAP score of 42 (IQR 36–48), the 106 patients enrolled were classified into High-KAP group (≥42, *n* = 53) and Low-KAP group (<42, *n* = 53). All multivariable regression analyses, however, used the continuous KAP score to avoid loss of statistical power and misclassification.

### 2.3 HRQoL measurement: FACT-bl

We used the FACT-Bl (version 4) to assess HRQoL. The FACT-Bl is a multidimensional, self-administered questionnaire that has been shown to be easy to administer, with acceptable internal consistency, test–retest reliability, and convergent and discriminant validity (1). The Chinese version of this instrument has been validated in a mainland Chinese bladder cancer population and demonstrated adequate reliability and validity (9). Furthermore, the Chinese version of this instrument has demonstrated adequate reliability and validity (9). Each item refers to the past 7 days; and is rated on a 5 point Likert scale ranging from 0 (“not at all”) to 4 (“very much”), with higher scores indicating better quality of life (15).

The FACT-Bl includes five subscales: Physical Well Being (PWB, 7 items, range 0–28), Social/Family Well Being (SWB, 7 items, 0–28), Emotional Well Being (EWB, 6 items, 0–24), Functional Well Being (FWB, 7 items, 0–28), and a Bladder Cancer Subscale (BlCS, 12 items, 0–48). The BlCS items cover disease specific concerns such as urinary control, frequency, dysuria, bowel function, weight loss, and body image. Certain items were reverse scored according to the official FACT-Bl scoring guidelines. The FACT-Bl total score is the sum of all five subscales (range 0–156) (16). In addition, we also calculated the TOI as the sum of PWB, FWB, and BlCS (range 0–104), which is a clinically relevant composite endpoint focusing on physical function, daily activities, and bladder specific symptoms (17).

All questionnaires were completed with assistance of trained research staff.

### 2.4 Covariates

We collected demographic and clinical characteristics as potential covariates in the multivariable analysis. Demographic variables included age, sex, body mass index (BMI, kg/m^2^), education level (categorized as junior high school or below, high school or vocational school, and college or above), and smoking status (current, former, or never smoker). Clinical variables comprised tumor stage (Ta, T1, or CIS), tumor grade (low or high), type of intravesical therapy (chemotherapy *vs.* BCG), and time after TURBT (months). These covariates were selected *a priori* based on previous studies demonstrating their independent associations with HRQoL in BC survivors, including age, sex, education, smoking, tumor stage, and treatment type (16, 18, 19).

**Figure 1:**
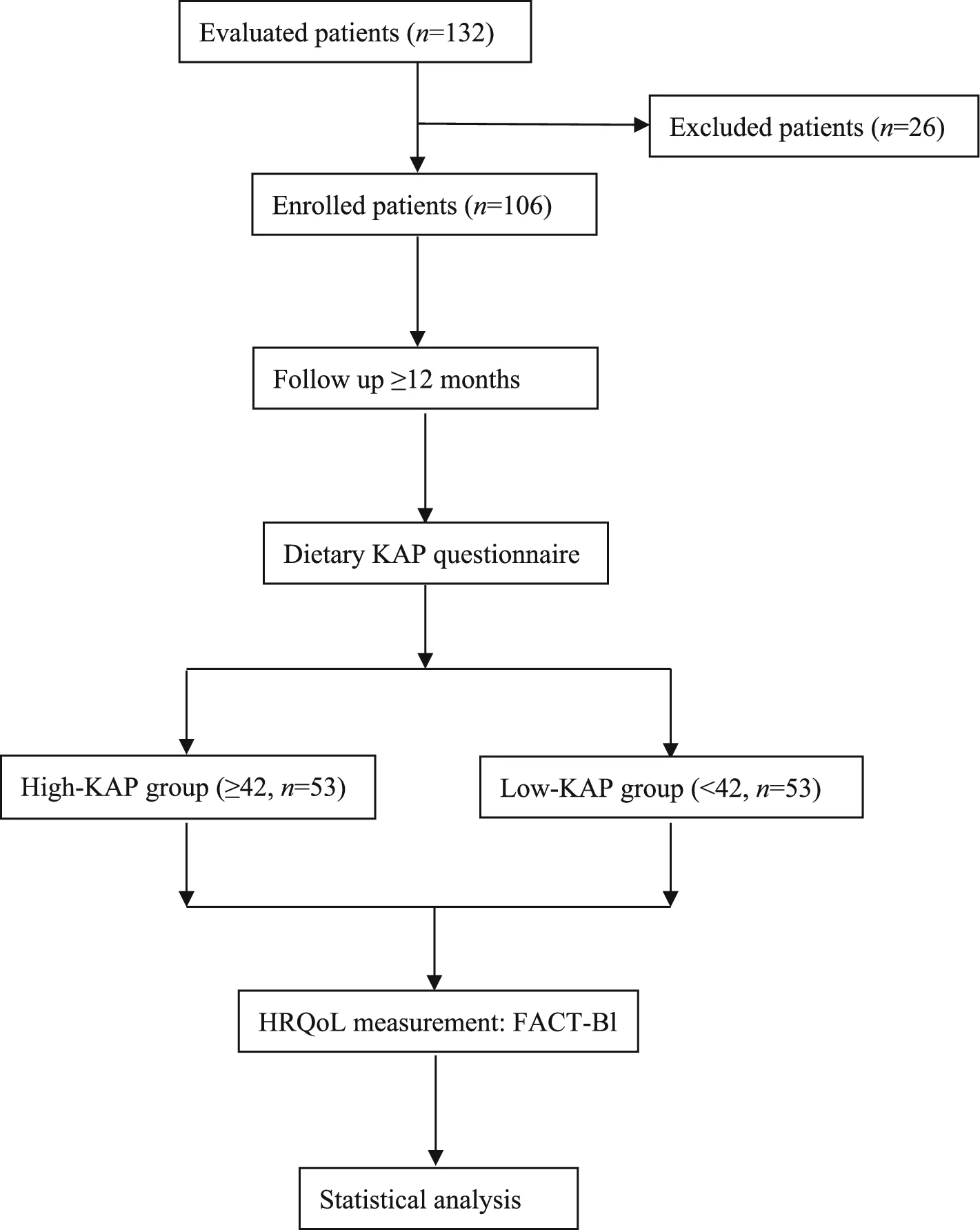
Flow chart of the study.

The study flow chart is seen in Figure 1.

### 2.5 Statistical analysis

All statistical analyses were performed using SPSS version 26.0 (IBM Corp., Armonk, NY, United States). Normality of continuous variables was assessed using the Shapiro Wilk test. Continuous variables following a normal distribution are described as mean ± standard deviation (SD), while those with a non-normal distribution are described as median with interquartile range (IQR). Categorical variables are presented as frequencies and percentages. Based on the distribution, continuous variables were compared using either the independent samples t test or the Mann Whitney U test, while categorical variables were compared using the chi square test. Effect sizes for group differences were reported as Cohen’s d.

Multivariable linear regression was used to examine the independent association between total KAP score (entered as a continuous variable in all primary models) and two outcomes: FACT-Bl total score (Model 1) and the TOI (Model 2). All models were adjusted for age, sex, education, smoking status, tumor stage, and type of intravesical therapy (chemotherapy vs. BCG). To identify which dimension of KAP drove the association, a separate model simultaneously included the knowledge, attitude, and practice subscale scores. Multicollinearity was assessed using the variance inflation factor (VIF), and all predictors had VIF values below 2.0. Regression assumptions (linearity, normality of residuals, and homoscedasticity) were checked using residual plots and the Shapiro–Wilk test on residuals, and were found to be adequately met. A two tailed *p* value <0.05 was considered statistically significant.

## 3 Results

### 3.1 Demographic and clinical characteristics of the participant

A total of 106 patients were included. The mean age was 67.5 years (SD 8.3; range 60–86), with 73.6% of the patients being male. Median time since TURBT was 15.0 months (IQR 12–18). Notably, the sample was limited to a relatively narrow survivorship window (12–18 months post-TURBT) due to the inclusion criterion of at least 12 months and the cross-sectional recruitment period. As for tumor characteristics, T1 stage accounted for 49.1% (52/106), Ta for 47.2% (50/106), and CIS for 3.8% (4/106). High grade tumors were present in 48.1% (51/106). Educational levels were as follows: most patients completed junior high school or below (62.3%, 66/106), and only 11.3% (12/106) held a college degree or higher, which was consistent with the characteristics of an elderly population in the local area.

All patients completed a full course of intravesical therapy as per protocol. The distribution of intravesical therapy types has been described in the Methods section (Section 2.1), with a *p* value of 0.899 between the two groups.

When comparing the High-KAP and Low-KAP groups, patients with higher KAP scores were more likely to be never smokers (69.8% *vs.* 50.9%, *p* = 0.022) and had a slightly higher proportion of college education (13.2% *vs.* 9.4%, *p* = 0.048). Other baseline characteristics, including age, sex, BMI, tumor stage, grade, as well as types of intravesical therapy, were well balanced between the two groups (Table 1).

**Table 1:** Baseline characteristics of patients by KAP groups.

Characteristic	High-KAP group (*n* = 53)	Low-KAP group (*n* = 53)	*p*-value
Age (years), mean ± SD	67.1 ± 8.6	67.9 ± 8.1	0.625
Male, *n* (%)	40 (75.5)	38 (71.7)	0.658
BMI (kg/m^2^), mean ± SD	24.3 ± 3.5	24.8 ± 3.9	0.487
Education, *n* (%)			0.048
Junior high school or below	30 (56.6)	36 (67.9)	
High school/vocational school	16 (30.2)	12 (22.6)	
College or above	7 (13.2)	5 (9.4)	
Smoking status			0.022
Current smoker	9 (17.0)	18 (34.0)	
Former smoker	7 (13.2)	8 (15.1)	
Never smoker	37 (69.8)	27 (50.9)	
Clinical T stage, *n* (%)			0.312
Ta	23 (43.4)	27 (50.9)	
T1	28 (52.8)	24 (45.3)	
CIS	2 (3.8)	2 (3.8)	
High-grade tumor, *n* (%)	26 (49.1)	25 (47.2)	0.841
Intravesical chemotherapy (MMC or Gem), *n* (%)	50 (94.3)	51 (96.2)	0.899
Intravesical BCG, *n* (%)	3 (5.7)	2 (3.8)	0.899
Time since TURBT (months), median (IQR)	15.5 (13–18)	14.5 (12–18)	0.568

### 3.2 Comparison of HRQoL scores between different dietary KAP groups

As shown in Table 2, the High KAP group scored significantly higher than the Low KAP group regarding PWB, FWB, BlCS, FACT-Bl total scores, and TOI score. The mean differences were large, with the TOI score difference of 15.6 points (95% CI 12.0–19.2, *p* < 0.001, Cohen’s d = 1.68). In contrast, there were no statistically significant differences between the two groups concerning SWB (20.0 ± 3.6 *vs.* 19.8 ± 3.8, *p* = 0.782) or EWB (18.5 ± 3.0 *vs.* 18.3 ± 3.1, *p* = 0.743). These findings indicated that higher dietary KAP scores were specifically associated with better physical, functional, and bladder specific quality of life, but not with social or emotional domains in this cohort.

**Table 2:** FACT-Bl scores and trial outcome index score by KAP groups.

Domain	High-KAP group (*n* = 53) (mean ± SD)	Low-KAP group (*n* = 53) (mean ± SD)	Mean difference (95% CI)	*P*-value	Cohen’s d
Physical well-being (PWB)	23.5 ± 3.1	20.1 ± 4.0	3.4 (2.0–4.8)	<0.001	0.95
Social/family well-being (SWB)	20.0 ± 3.6	19.8 ± 3.8	0.2 (−1.2–1.6)	0.782	0.05
Emotional well-being (EWB)	18.5 ± 3.0	18.3 ± 3.1	0.2 (−1.0–1.4)	0.743	0.07
Functional well-being (FWB)	22.5 ± 3.2	17.8 ± 4.1	4.7 (3.2–6.2)	<0.001	1.27
Bladder cancer-specific (BlCS)	38.1 ± 5.2	30.6 ± 6.3	7.5 (5.1–9.9)	<0.001	1.29
FACT-Bl total score	115.4 ± 12.6	94.2 ± 14.8	21.2 (16.4–26.0)	<0.001	1.54
Trial outcome index (TOI)	84.1 ± 8.5	68.5 ± 9.9	15.6 (12.0–19.2)	<0.001	1.68

### 3.3 Independent association between total dietary KAP score and HRQoL

After adjusting for age, sex, education, smoking status, tumor stage, and type of intravesical therapy, the result showed that total dietary KAP score (as a continuous variable) remained a strong independent predictor of both FACT-Bl total score and TOI score (Table 3). Specifically, each one-point increase in total KAP score was associated with a 0.58-point increase in FACT-Bl total score (95% CI: 0.42–0.74; standardized *β* = 0.51; *p* < 0.001), with the model explaining 59% of the variance (adjusted *R*^2^ = 0.59). Similarly, for TOI, the regression coefficient was 0.62 (95% CI: 0.48–0.76; standardized *β* = 0.57; *p* < 0.001; adjusted *R*^2^ = 0.61). Among the covariates, current smoking was negatively associated with FACT-Bl total score (*β* = −7.30, *p* = 0.008), while college education remained positively associated (*β* = 6.20, *p* = 0.012). The type of intravesical therapy (chemotherapy *vs.* BCG) was not a significant predictor (*p* = 0.68).

**Table 3:** Multivariable linear regression analyses of factors associated with HRQoL outcomes.

Variable	Model 1:outcome = FACT-Bl total score			Model 2: outcome = Trial outcome index (TOI)		
	*β* (95% CI)	Standardized *β*	*P*-value	*β* (95% CI)	Standardized *β*	*P*-value
Total KAP score (per 1-point increase)	0.58 (0.42, 0.74)	0.51	<0.001	0.62 (0.48, 0.76)	0.57	<0.001
Current smoker (*vs.* never smoker)	−7.30 (−12.50, −2.10)	−0.22	0.008	−5.10 (−9.80, −0.40)	−0.18	0.034
College or above (*vs.* ≤ junior high school)	6.20 (1.40, 11.00)	0.19	0.012	3.90 (−0.80, 8.60)	0.14	0.098
Intravesical therapy (BCG *vs.* chemotherapy)	−1.80 (−8.20, 4.60)	−0.05	0.68	−1.20 (−6.50, 4.10)	−0.04	0.71
Age (per year)	−0.10 (−0.35, 0.15)	−0.06	0.42	−0.08 (−0.30, 0.14)	−0.05	0.48
Sex (male *vs*. female)	1.20 (−2.60, 5.00)	0.04	0.53	0.90 (−2.40, 4.20)	0.04	0.59
Tumor stage (T1 *vs.* Ta)	−1.10 (−4.20, 2.00)	−0.05	0.49	−0.70 (−3.30, 1.90)	−0.04	0.60
High-grade tumor (*vs.* low-grade)	−0.50 (−3.80, 2.80)	−0.02	0.77	−0.30 (−3.10, 2.50)	−0.02	0.83
Model adjusted *R*^2^	—	0.59	—	—	0.61	—

Sensitivity analysis using the KAP total score as a continuous variable without dichotomization confirmed the robustness of these findings, with consistent effect sizes and significance levels (data not shown).

### 3.4 Associations of dietary KAP subdomains with HRQoL

To identify which domain of dietary KAP contributed most to the association with HRQoL, the scores of knowledge, attitude, and practice subdomains were simultaneously entered into multivariable linear regression models. After adjustment for age, sex, education, smoking status, tumor stage, and type of intravesical therapy, the result showed that only the practice subdomain remained an independent significant predictor for both FACT-Bl total score and TOI score (Table 4).

**Table 4:** Multivariable linear regression analysis of KAP subdomains associated with HRQoL outcomes.

Variable	Model 1: FACT-Bl total score			Model 2: Trial Outcome Index (TOI)		
	*β* (95% CI)	Standardized *β*	*P*-value	*β* (95% CI)	Standardized *β*	*P*-value
Knowledge	0.26 (−0.20, 0.72)	0.09	0.24	0.23 (−0.25, 0.71)	0.08	0.31
Attitude	0.18 (−0.09, 0.45)	0.11	0.18	0.15 (−0.13, 0.43)	0.09	0.26
Practice	1.24 (0.87, 1.61)	0.55	<0.001	1.31 (0.94, 1.68)	0.59	<0.001
Model adjusted *R*^2^	—	0.62	—	—	0.64	—

Adjusted for: age, sex, education, smoking status, tumor stage, type of intravesical therapy.

Each 1-point increase in practice score was associated with a 1.24-point increase in FACT-Bl total score (95% CI: 0.87–1.61; *p* < 0.001). By contrast, the knowledge subdomain (*p* = 0.24) and attitude subdomain (*p* = 0.18) showed no independent association. For TOI, practice was also the only significant predictor, with a regression coefficient of 1.31 (95% CI: 0.94–1.68; *p* < 0.001).

These findings indicated that actual dietary practice, rather than knowledge or attitude alone, was the key determinant associated with better quality of life in this study population.

## 4 Discussion

In this cross-sectional, real-world study of 106 elderly recurrence-free NMIBC patients at 12 months following TURBT, our results demonstrated that higher dietary KAP scores were independently and strongly associated with better HRQoL. As shown in Table 2, the High-KAP group scored significantly higher than the Low-KAP group in PWB, FWB, BlCS, FACT-Bl total score, and TOI. The between-group differences presented large effect sizes, indicating clinically meaningful disparities. The TOI differed by 15.6 points (95% CI 12.0–19.2, *p* < 0.001, Cohen’s d = 1.68). Notably, after multivariable adjustment, only the dietary practice subdomain remained an independent predictor of HRQoL, whereas knowledge and attitude showed no independent effect. However, due to the cross-sectional design, these associations should not be interpreted as causal. The observed relationships may be subject to reverse causality (e.g., patients with better physical function may find it easier to adhere to healthy diets) and residual confounding from unmeasured variables. This finding highlights that translating dietary knowledge and positive attitudes into actual daily behaviors is critical for enhancing physical well-being, daily functional status, and bladder-specific quality of life in this survivorship population.

**Figure 2:**
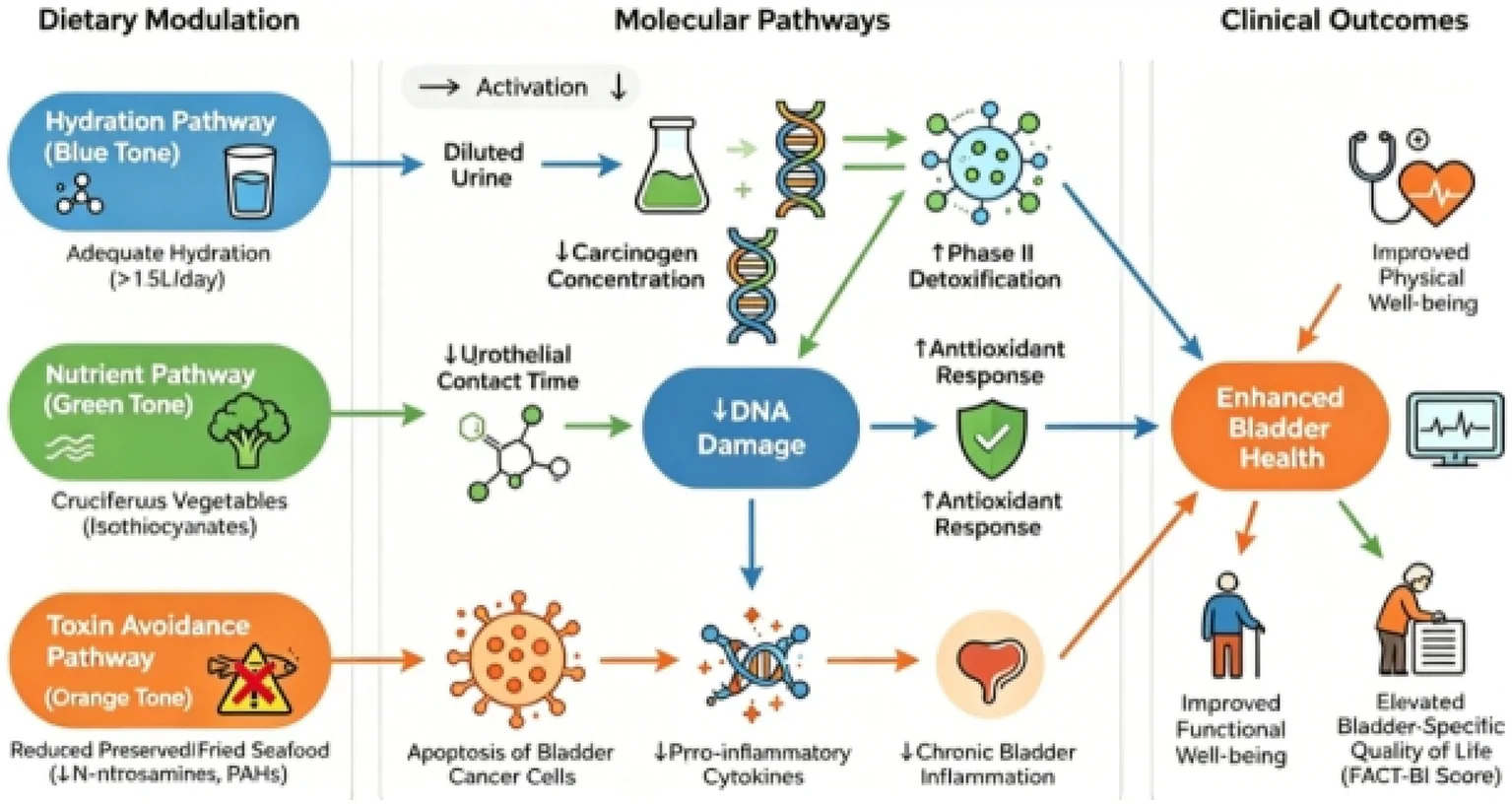
Proposed mechanistic pathway linking dietary practices to health-related quality of life (HRQoL) in elderly non-muscle-invasive bladder cancer (NMIBC) patients. The arrow also indicates the reverse pathway, i.e., better HRQoL may in turn facilitate adherence to healthy dietary practices. This figure was generated using Doubao (ByteDance) and input prompts can be found in Supplementary File 1.

Several biologically plausible pathways may underlie the observed associations, although none of these pathways were directly tested in this study. The “dilution hypothesis” has long been proposed to explain how adequate fluid intake could protect the bladder mucosa: higher urinary volume reduces both the concentration of potential carcinogens and the duration of their contact with the urothelium, thereby mitigating cumulative DNA damage and chronic inflammation (20, 21). This mechanism is directly relevant to patient’s quality of life, as frequent voiding of dilute urine might alleviate irritative bladder symptoms such as dysuria and urinary frequency, which are involved in the BC subdomain of FACT-Bl. Cruciferous vegetables (22) broccoli, cabbage, bokchoy are rich in glucosinolates that hydrolyse to bioactive isothiocyanates (ITCs), such as sulforaphane (23). ITCs are known to induce phase II detoxification enzymes, promote apoptosis of BC cells, and exert both anti-inflammatory and antioxidant effects, subsequently reducing treatment-related discomfort and improving physical well-being. Indeed, clinical studies have shown that raw cruciferous vegetable intake is inversely associated with BC risk and mortality (24). Conversely, preserved seafood a traditional staple in coastal Zhejiang contains N-nitrosodimethylamine and other volatile N-nitrosamines, which have been detected at notable levels in salted and dried local products. These compounds can induce oxidative DNA damage and sustained epithelial irritation. Moreover, patients who frequently consume deep-fried or grilled fish may be exposed to additional mutagens, including heterocyclic amines and polycyclic aromatic hydrocarbons. The latters are formed during high-temperature cooking (25). It should be noted that these pathways are primarily relevant to HRQoL improvement rather than to recurrence prevention, which involves distinct carcinogenic mechanisms. These mechanisms are speculative and require future mechanistic or interventional studies to confirm. Taken together, these biological pathways help explain why better dietary practice adequate hydration, regular intake of cruciferous vegetables, and limited consumption of preserved or fried seafood-is associated with higher physical well-being, functional well-being, and bladder-specific quality of life scores in our patients. A proposed mechanistic pathway underlying our findings is shown in Figure 2.

Our finding that practice not knowledge or attitude independently correlates with HRQoL has important pragmatic implications. Elderly survivors often possess adequate nutritional knowledge but face functional, economic, and social barriers to implementation (6). Therefore, purely educational approaches may be insufficient. Clinicians should prioritize action oriented, low threshold behavioral goals, such as setting daily water intake targets, encouraging specific vegetable portions, and reducing preserved seafood frequency, rather than providing abstract dietary guidelines. This aligns with the chronic care model that emphasizes self-management support tailored to the patient’s daily context.

In the clinical practice of our region, the actual use of BCG is restricted due to several concerns, such as its side effects (bladder irritation, frequent and painful urination, hematuria, and fever) (26), BCG shortage, higher costs (as it is fully self-funded in China), and patient preferences, although current guidelines recommend intravesical BCG as the preferred option for intermediate and high-risk NMIBC. Consequently, intravesical chemotherapy (MMC or Gem) was the dominant treatment regimen in our cohort (95.3% of participants), in parallel with the real-world scenario in many Chinese regional hospitals (27). Notably, despite the low adoption rate of BCG (only 4.7%), multivariate adjustment showed that treatment type (chemotherapy *vs.* BCG) was not significantly correlated with either FACT-Bl total score or TOI (*p* > 0.05), suggesting that the observed variation in HRQoL was predominantly attributable to differences in individual dietary practice rather than the type of intravesical therapy.

Moreover, our findings extend prior research in several important ways, particularly by focusing on older adults with NMIBC a population for whom HRQoL is both especially vulnerable and clinically consequential. Previous studies using the SEER-MHOS linked data have shown that older BC survivors experienced significant declines in physical quality of life following diagnosis, and poorer physical quality of life was independently associated with shorter overall survival (28, 29). However, most of such works were focused on oncologic outcomes or broad HRQoL comparisons, leaving a gap between these established endpoints and the modifiable behavioral factors such as diet (30). Our study filled this gap by applying a culturally adapted dietary KAP questionnaire to a cohort of older, recurrence-free NMIBC patients. Importantly, the large between-group differences we found in FACT-Bl total and TOI scores were comparable in magnitude to the HRQoL deficits reported in older cancer survivors with multiple comorbidities or functional limitations (3). Furthermore, while cross-sectional studies in general older populations have linked healthier dietary patterns to better self-rated health (31), our findings provided disease-specific evidence that such associations hold true in elderly BC survivors, even after adjusting for tumor stage and treatment. This finding is clinically relevant, as older patients typically prioritize HRQoL over longevity in treatment decisions yet their dietary habits remain largely overlooked in routine survivorship care.

The finding that knowledge and attitude were not independently associated with HRQoL after adjustment has clear implications for the care of older NMIBC patients. Elderly individuals often face unique barriers to translating health knowledge into daily behavior: age-related declines in physical function, multiple chronic conditions, social isolation, and economic constraints might all limit their ability to modify dietary habits (6). Indeed, clinical studies in older adults with chronic diseases have shown that simply providing information is insufficient. Instead, sustained improvements in HRQoL require practical, action-oriented support (31, 32). Our results were in agreement with previous literature (32), and further suggested that for elderly BC survivors, dietary interventions should move beyond education alone. Rather than emphasizing abstract nutritional knowledge, clinicians and nurse educators might concentrate on concrete, feasible behavioral goals, for example, drinking water at least 1.5 liters daily, consuming cruciferous vegetables at least twice weekly, and reducing the frequency of eating preserved seafood. Considering that older BC patients are more likely to have multiple comorbidities and functional limitations (29), and that physical HRQoL strongly predicts survival (28, 33), integrating such dietary practices into daily life could represent a low-cost, high-impact intervention to improve both quality of life and long-term outcomes in the elderly population. Nutritional challenges in older adults extend beyond knowledge deficits. Age-related changes in taste, dentition, gastrointestinal motility, and polypharmacy can all impede dietary modification (34). Our findings highlight the need for individualized nutritional assessment that considers frailty, functional status, and socioeconomic resources factors not captured in the current study.

Several limitations should be noted in this study. First, the cross-sectional design precludes any causal inference between dietary KAP and HRQoL. Therefore, the observed associations should be interpreted as correlational rather than causal, and the observed associations may also reflect reverse causality, whereby better HRQoL enables healthier eating. Second, both dietary KAP and HRQoL were assessed using self-reported questionnaires, which might introduce recall bias and social desirability bias, despite the use of trained research staff to assist investigation. Third, this was a single center study carried out in a coastal city of Zhejiang Province, China, where dietary habits (e.g., high consumption of preserved seafood) and clinical practices (e.g., predominant use of intravesical chemotherapy) may not be representative of other regions or other countries, thereby limiting the generalizability of the findings. Fourth, although we adjusted for several important covariates, potential confounders such as physical activity, social support, economic status, and comorbidity burden were not evaluated; these factors have been shown to independently influence HRQoL in cancer patients and might partly explain the observed associations. Such aspects will be further investigated in future studies. Fifth, the use of median dichotomization for descriptive comparisons, while clinically interpretable, may reduce statistical power and introduce misclassification; however, our primary analyses used continuous KAP scores and confirmed the main findings. Sixth, due to the inclusion criterion of at least 12 months post-TURBT, our sample was concentrated in the 12–18 month survivorship window, which may limit applicability to longer-term survivors. Seventh, the KAP questionnaire, though internally consistent and face-valid, lacks formal construct validation (e.g., confirmatory factor analysis) in this specific elderly NMIBC population. Finally, the overwhelming use of intravesical chemotherapy (95.3%) limits applicability to BCG predominant settings. Future prospective, multi-centered studies with larger sample sizes, and longer follow-up periods are warranted to confirm our findings, and to test the proposed mechanistic pathways linking dietary practices to HRQoL in elderly NMIBC survivors.

## 5 Conclusion

In this cross-sectional, survey cohort of elderly recurrence-free NMIBC patients at 12 months after TURBT, higher dietary KAP scores are associated with better HRQoL, and this association appears to be primarily attributable to the practice domain rather than knowledge or attitude alone. These findings support the integration of behavior-focused, culturally adapted dietary support into survivorship care, although future prospective studies are needed to establish causality and evaluate the effectiveness of specific dietary interventions.

## Data Availability

The original contributions presented in the study are included in the article/Supplementary material, further inquiries can be directed to the corresponding author.

## Glossary

BC: Bladder cancer
NMIBC: Non-muscle-invasive bladder cancer
TURBT: Transurethral resection of bladder tumor
HRQoL: Health-related quality of life
KAP: Knowledge-attitude-practice
SF-36: Medical Outcomes Study 36-Item Short Form
EORTC QLQ-NMIBC24: European Organization for Research and Treatment of Cancer Quality of Life Questionnaire-NMIBC24
BCI: Bladder Cancer Index
FACT-Bl: Functional Assessment of Cancer Therapy-Bladder Cancer
TOI: Trial Outcome Index
BCG: Bacillus Calmette-Guérin
MMC: Mitomycin C
Gem: Gemcitabine
CIS: Carcinoma in situ
IQR: Interquartile range
PWB: Physical Well-Being
SWB: Social/Family Well-Being
EWB: Emotional Well-Being
FWB: Functional Well-Being
BlCS: Bladder Cancer Subscale
BMI: Body mass index
SPSS: Statistical Product and Service Solutions
SD: Standard deviation
VIF: variance inflation factor
ITCs: Isothiocyanates
PAHs: Polycyclic aromatic hydrocarbons
SEER-MHOS: Surveillance, Epidemiology, and End Results-Medicare Health Outcomes Survey
