# Supplementary material for: Association of dietary practice, rather than knowledge or attitude, with health-related quality of life in elderly NMIBC survivors: a cross-sectional study
Source: Front Nutr. 2026 Aug 27;13:1918491. doi: 10.3389/fnut.2026.1918491 (PMC13557947; doi:10.3389/fnut.2026.1918491)
Supplement: Supplementary file 1 [file Supplementary_file_1.docx]

The Input prompts for Figure 2 (generated using Doubao (ByteDance)) are as follows:

Create an infographic illustrating three dietary modulation pathways (hydration, nutrient intake, and toxin avoidance) and their downstream molecular effects (reduced DNA damage, enhanced detoxification, and suppressed inflammation) that collectively improve bladder health and lead to better physical, functional, and bladder‑specific quality of life in elderly non‑muscle‑invasive bladder cancer survivors. Use clear icons and arrows to show the causal flow from dietary practices to clinical outcomes.
